# The carbon footprint of dietary guidelines around the world: a seven country modeling study

**DOI:** 10.1186/s12937-021-00669-6

**Published:** 2021-03-01

**Authors:** Brittany Kovacs, Lindsey Miller, Martin C. Heller, Donald Rose

**Affiliations:** 1 Foundation for International Medical Relief of Children, Philadelphia, PA 19102 USA; 2 Healthy Eating Research, Duke Global Health Institute, Duke University, Durham, NC 27708 USA; 3 Center for Sustainable Systems, School for Environment & Sustainability, University of Michigan, Ann Arbor, MI 48109 USA; 4 Tulane Nutrition, School of Public Health and Tropical Medicine, Tulane University, New Orleans, LA 70112 USA

**Keywords:** Food-based dietary guidelines, Global warming, Greenhouse gas emissions, Carbon footprint, dataFIELD, FAO food balance sheet

## Abstract

**Background:**

Do the environmental impacts inherent in national food-based dietary guidelines (FBDG) vary around the world, and, if so, how? Most previous studies that consider this question focus on a single country or compare countries’ guidelines without controlling for differences in country-level consumption patterns. To address this gap, we model the carbon footprint of the dietary guidelines from seven different countries, examine the key contributors to this, and control for consumption differences between countries.

**Methods:**

In this purposive sample, we obtained FBDG from national sources for Germany, India, the Netherlands, Oman, Thailand, Uruguay, and the United States. These were used to structure recommended diets using 6 food groups: protein foods, dairy, grains, fruits, vegetables, and oils/fats. To determine specific quantities of individual foods within these groups, we used data on food supplies available for human consumption for each country from the UN Food and Agriculture Organization’s food balance sheets. The greenhouse gas emissions (GHGE) used to produce the foods in these consumption patterns were linked from our own database, constructed from an exhaustive review of the life cycle assessment literature. All guidelines were scaled to a 2000-kcal diet.

**Results:**

Daily recommended amounts of dairy foods ranged from a low of 118 ml/d for Oman to a high of 710 ml/d for the US. The GHGE associated with these two recommendations were 0.17 and 1.10 kg CO_2_-eq/d, respectively. The GHGE associated with the protein food recommendations ranged from 0.03 kg CO_2_-eq/d in India  to 1.84 kg CO_2_-eq/d in the US, for recommended amounts of 75 g/d and 156 g/d, respectively. Overall, US recommendations had the highest carbon footprint at 3.83 kg CO_2_-eq/d, 4.5 times that of the recommended diet for India, which had the smallest footprint. After controlling for country-level consumption patterns by applying the US consumption pattern to all countries, US recommendations were still the highest, 19% and 47% higher than those of the Netherlands and Germany, respectively.

**Conclusions:**

Despite our common human biology, FBDG vary tremendously from one country to the next, as do the associated carbon footprints of these guidelines. Understanding the carbon footprints of different recommendations can assist in future decision-making to incorporate environmental sustainability in dietary guidance.

**Supplementary Information:**

The online version contains supplementary material available at 10.1186/s12937-021-00669-6.

## Background

More than 90 countries have developed food-based dietary guidelines, which provide brief science-based messages to promote nutritious diets [1]. Previous research comparing FBDG among countries has traced the development or implementation of the guidelines [2–4] or analyzed the pictorial messages associated with the FBDG [5–11]. Other studies have focused on qualitative comparisons of countries’ FBDG by investigating similarities and differences in key messages, the number of food groups included, the categorization of foods into food groups, and recommended serving sizes [4, 10–15].

A recent comparative analysis of key messages in FBDG looked at the inclusion of messages promoting sustainability [16]. Four of 83 countries included in the study had key messages related to sustainability, such as eating more plant-based foods and reducing meat consumption, integrated into their official FBDG. Besides these four countries, additional countries have addressed sustainability in quasi-official guidelines or through recommendations made by other government-funded entities [16], yet sustainability considerations and recommendations have not been integrated into the majority of official FBDG around the world.

Addressing sustainability in FBDG will become critical as the global population continues to grow towards an estimated 9.8 billion people by 2050 [17] and as climate change threatens the world’s food supply and global food security [18]. A number of changes in the climate system, including increasing temperatures, warming oceans, rising sea levels, and melting ice sheets, have been observed over the past several decades [18]. Many of these changes are associated with increased levels of anthropogenic greenhouse gas emissions (GHGE), which are continuing to rise [18]. Food systems are estimated to contribute up to 29% of global anthropogenic GHGE [19], and agriculture is one of the largest contributors of GHGE within food systems, with livestock responsible for approximately 14.5% of total global anthropogenic GHGE [20]. The GHGE contribution of the agricultural sector is expected to continue to rise [21] as urbanizing populations around the world continue to shift to diets higher in animal products and lower in legumes, coarse grains, and other vegetables [22]. The dual burden of recent global dietary change on health and the environment presents a global challenge [21, 23].

One solution to address both health and environmental concerns is to shift current dietary patterns to diets that are both nutritious and sustainable. Though dietary shifts may require interventions on multiple levels, including macro-level policy changes and micro-level behavior change, a simple macro-level intervention is for governments to promote diets that are both nutritious and sustainable and incorporate these recommendations into nutrition and food-related policies. Although many countries have already developed FBDG to promote a culturally relevant, nutritious diet and to address nutrition-related public health concerns, more work is needed on incorporating environmental sustainability, such as dietary impacts on GHGE, into countries’ FBDG.

Previous single-country studies comparing the carbon footprint of recommended diets to current average population intakes in Europe and Australia have largely found that shifting from the average national diet to the diet recommended by the country’s FBDG would lower GHGE [24–27]. Several multi-country studies have been conducted that also tend to show reduced GHGE impacts of diets based on recommendations as compared to average diets [28–30]. However, these reductions have not been seen in other studies comparing current eating patterns to guidelines in the United States [31–34].

These results suggest there may be differences in the carbon footprint of recommended dietary patterns among countries, in particular, with the footprint of the US guidelines being higher than other countries. Unfortunately, single-country studies do not allow for evaluation of the carbon footprint differences in the recommendations between countries. Moreover, the multi-country studies, which could allow for comparisons using a consistent methodology across countries, use a country’s current eating pattern as part of the specification of the recommended diet. Analytically this is necessary, since FBDG are never specific enough to calculate a footprint without assuming a baseline food pattern. But this implies that comparisons between FBDG footprints are dependent on the countries’ existing food consumption pattern.

Here we address this analytic gap in the literature by developing a simple method to make comparisons between FBDG of different countries, while “controlling” for a country’s consumption patterns. We are particularly interested in guidelines from the US because of the anomaly discussed above, but also because the country is the world’s second largest contributor to greenhouse gas emissions [35] and there has been recent controversy about the exclusion of sustainability considerations from these guidelines [36, 37]. Here, we use a purposive sample of countries that includes the US and 6 others to examine this issue. Specifically, this study aims to: 1) describe the differences in an illustrative sample of different countries’ recommended diets; 2) develop a method to compare the GHGE of FBDG using food supply data and a database of global warming impacts; and 3) examine the differences in global warming impacts (i.e. GHGE, or carbon footprint) of these countries’ recommended diets with a method that uses countries’ existing consumption patterns, as well as one that controls for differences in these patterns.

## Methods

### Study design and sample

We used purposive sampling to include the US and a diverse range of countries that would shed light on the variation in potential impacts of FBDG. We relied on the United Nations Food and Agriculture Organization’s (FAO) online repository of FBDG [1] to examine a list of such countries. Only FBDG available in English or Spanish were considered for inclusion in this study, as were those with a list of clearly defined food groups (e.g. grains) and daily quantitative recommendations for each food group, including defined weight- or volume-based serving sizes, which are needed to model greenhouse gas emissions. We selected two European countries, Germany and the Netherlands, for which earlier research had shown that diet shifts towards their guidelines would lower GHGE [26, 27, 38]. To seek maximum variation in GHGE, we chose India [39] and the US vegetarian FDBG [40], which do not include meat in their FBDG, since animal proteins are a major dietary contributor to this impact. Other countries, including Oman [41], Thailand [42], and Uruguay [43] were chosen for diverse geographic representation. Additional countries, including Australia, Belize, Malta, and Malaysia were considered, but they did not have either specific quantitative recommendations for all food groups (Malta) or defined weight- or volume-based serving sizes for all food groups (Australia, Belize, Malaysia). Finally, we also included the EAT-Lancet dietary recommendations, since these are global guidelines explicitly designed with environmental sustainability in mind [44].

### Food-based dietary guidelines (FBDG)

In addition to the FAO repository, we used government websites to obtain additional details on country-level FBDG. Quantitative recommendations for discrete food groups were included in the analysis. “Discretionary calories” were included in the analysis based on each country’s recommendations. For example, Germany, India, and Uruguay specified additional intakes of sugar, so we included those quantities in our analysis. The US and the Netherlands included discretionary calorie amounts, but no specificity on the composition of those calories, so we increased GHGE to cover the discretionary calories assuming the same composition as the rest of the recommended diet. We did not include non-caloric items, such as coffee or tea, since there were no specific recommendations for them. Additionally, qualitative recommendations (e.g. “eat a variety of food to ensure a balanced diet”) were not included in this analysis. For consistency, the main dietary pattern equal or closest to 2000 kcal was selected from each country’s FBDG, regardless of the targeted age-sex group. If the main pattern was higher, as in the EAT-Lancet recommendation of 2500 kcal, or the Netherlands’ recommendation of 2053, or Uruguay’s recommendation of 2200 kcal, quantities were scaled to 2000 kcal. If recommended quantities of food groups were only listed in ranges, we used the mid-point of those ranges for our analysis, otherwise we used the specified amounts.

### FAO food balance sheet data

Although the FBDG used in this study provide daily quantitative recommendations for clearly defined food groups, the guidelines do not provide recommendations for the specific foods within each group. For example, Thailand recommends 135 g/d of protein foods, but does not make further recommendations on how much beef, pork, or chicken should be consumed. There is tremendous variation in the global warming impacts of these individual foods [45], so item specificity within a food group is needed. To address this, we used national consumption patterns of specific foods within each food group. We relied on FAO food balance sheets to obtain these country-level patterns, specifically relying on the annual per capita food supply to humans. This is ‘apparent consumption,’ since it is obtained by summing the contributions to total domestic food availability (production, imports, and stock changes) and subtracting exports and other foods utilized for non-human purposes (feed, seed, processing, waste, exports, and other uses). The 2013 food supply quantity data (kilograms/capita/year) for 58 commodities in the protein foods, grains, fruit, vegetables, and oils/fats food groups in seven countries were used in this study [46]. The FAO food balance sheets did not provide sufficient data on dairy products for this analysis, so we used the 2013 Organization for Economic Co-operation and Development (OECD)-FAO Agricultural Outlook dairy data [47] in the same manner as the FAO food balance sheets for four types of dairy products.

Apparent consumption patterns were described by a series of proportions, that is, the food supply quantity of an individual food item (e.g. poultry) divided by the sum of quantities of all items in a food group (e.g. protein foods). These consumption patterns were different for each country and not all countries consumed all items in a group. Prior to calculating these apparent consumption patterns, commodity weights were converted to cooked edible weights. This required a series of factors to convert individual protein foods (i.e. meats, poultry, and fish) from carcass weight to raw, boneless weight [45] and then again to cooked, boneless weight using an average of dry and wet cooking method conversion factors [48, 49]. Conversion factors were also applied to remove the shells from groundnuts, nuts and products, eggs, molluscs, and crustaceans [45, 48] and also to convert legumes and grains from raw weight to cooked weight [50].

### Environmental impact data: dataFIELD

Data on the greenhouse gas emissions (GHGE) for the production of different foods come from the database of Food Impacts on the Environment for Linking to Diets (dataFIELD), which is based on an extensive review of the life cycle assessment literature [45]. Articles and reports available in the public domain and published in English from 2005 to 2016 were included in this review, with cradle-to-farm gate impact factors used for the vast majority of the 332 commodities in this database. DataFIELD includes studies throughout the world, and although differences in production practices affect GHGE values, there is not sufficient research to identify GHGE values of foods disaggregated to the country level. Thus, we used the same mean GHGE values, expressed as carbon dioxide equivalents per kilogram of food (CO_2_-eq/kg), for all countries studied here, and applied them to 58 FAO commodities and four OECD-FAO dairy products. Aggregate values were created for certain commodities that included multiple foods (e.g. “nuts and products”, “freshwater fish”). GHGE data were not available for five other commodities and were excluded from our analysis. These commodities – cephalopods, aquatic animals other than fish or seafood, sorghum, palm kernel oil, and rice bran oil – contributed a trivial amount to consumption patterns, accounting for less than 10 kcal per capita per day, on average.

### Carbon footprint calculations

To calculate the GHGE for each country’s FBDG recommendation for each food group ‘g’, we calculated a weighted average of the GHGE for the specific commodities in that group and multiplied it by the recommended daily amount of that group using the following formula:

$$ {GHGE}_g={\sum}_{c=1}^C{REC}_g\times {Proportion}_c\times {CF}_c\times {GHGE}_c $$

where *REC*_*g*_ is each country’s FBDG recommended daily amount of group g, for example, 2 cups of fruit. *Proportion*_*c*_ is the quantity share of each commodity c consumed out of the total food group quantity, for example, the proportion of oranges out of total fruits consumed, which comes from food balance sheet data for each country. *CF*_*c*_ is a conversion factor for adjusting the recommendation to kilograms of commodity c, and *GHGE*_*c*_ is the greenhouse gas emissions (kg CO_2_-eq) to produce a kilogram of this commodity. For the EAT-Lancet guidelines, the same procedure was followed using the apparent consumption pattern of the US. Finally, the GHGE of all food groups within a country were summed for the total GHGE that could be attributed to eating a diet as recommended by the FBDG of a particular country.

Conversion factors were used in several ways in the final calculations. For the FBDG that provided recommendations in units of weight other than grams (e.g. ounces), conversions were made using the standard conversion factor for ounces to grams. For recommendations given by volume (e.g. cups), we used food composition tables [50] that provided the necessary conversions to grams of an equivalent amount of food in calories. For example, a cup of black beans has 662 kcal and 100 g of black beans has 341 cal, so a cup of black beans would be equivalent to 194 g. For Thailand’s recommendations, which define one recommended serving of fruit as a “piece” of fruit, one piece of fruit was estimated to be equivalent to one cup of fruit. If the commodity group was aggregated, an average equal calorie conversion was calculated based on the individual foods included in that commodity group. Some commodities, including meat, fish, grains and legumes, were converted from cooked weights (used in the recommendations) to raw, edible weights (used in the global warming impact factors). Grains and legumes were converted using equal calorie conversion factors, and meat and fish and seafood were converted using an average of dry and wet cooking method conversion factors [49].

### Determining differences in FDBG ‘controlling’ for national dietary patterns

Since country recommendations do not specify food items within a group, we used FAO food balance sheet data for each country to calculate these specific food consumption patterns, as described above. This means that differences in the carbon footprints between countries are partly due to each country’s current consumption pattern, as reflected in these FAO data. To control for this effect, we recalculated each country’s FBDG carbon footprint using the same food consumption pattern across all countries, that of the United States. At times, this required different aggregations of foods. For example, the protein foods group in India’s FBDG does not include meats, so we only considered plant protein foods – beans, peas, and legumes – when applying the US consumption pattern to the India FBDG.

## Results

Although all the FBDG used in this analysis are designed to meet the nutritional requirements of adults, variation was found in the quantitative daily recommendations presented in the FBDG. For presentation purposes, all FBDG were organized into six major food groups – protein foods, dairy, grains, fruits, vegetables, and oils/fats. Recommended daily quantities for each group for each FBDG are presented in Table 1, summed into a single metric (g or ml) and scaled to a 2000-kcal diet for comparison purposes. For protein foods, this ranges from 75 g in India to 156 g in the US to 168 g for Oman, though the latter is more than half vegetable protein. For dairy foods, recommended daily amounts range from 118 ml for Oman to 710 ml for the US. The composition of these recommended food groups varies widely; roots and tubers, potatoes, legumes, and pulses are often grouped in different food groups among the seven countries, and the composition of the protein foods group also differed. Our display of groups in Table 1 reflects the way that the majority of countries structured their FBDG. For this reason, and because of its potential substitutability for animal proteins, we included legumes and pulses within the protein foods group. Some countries make specific recommendations within the larger recommendations for food groups, as India does within the vegetable food group, or as Germany or the EAT-Lancet pattern do with the protein foods group. See Supplemental Table 1 for the detailed recommendations expressed in each country’s FBDG.

**Table 1:** Daily recommended amounts of each food group for a 2000-kcal diet by country, scaled to common units^**a**^

	Protein foods (g)	Dairy^**b**^ (ml)	Grains (g)	Fruit (g)	Vegetables (g)	Oils/fats (g)
Germany^**c**^	99	524	362	250	512	35
India^**c**^	75^**e**^	300	330	100	500	25
Oman	168	118	662^**g**^	686	420	56
The Netherlands^**d**^	150	415	428^**g**^	200	250	40
Thailand	135	237	600^**h**^	784	200	N/A
United States^**d**^	156	710	170	392	350	27
US Vegetarian^**d**^	97	710	184	392	350	27
Uruguay^**c**^	91^**f**^	455	227^**1**^	455^**j**^		25
EAT-Lancet	167	194	186	160	280	42

^**a**^Daily recommendations for a 2000-kcal diet. For the Netherlands, Uruguay, and EAT-Lancet, the 2053 kcal, 2200 kcal and 2500 kcal diets were scaled to 2000 kcal. For recommendations that are presented as ranges, we used the mid-points of the range. See Supplemental Table 1 for more details on the specific recommendations from each country.

^**b**^Dairy quantities are listed as volumes. When the FBDG specified a gram amount for milk, this was converted to ml using a density of 1.0305 g/ml [54]

^**c**^These diets also include recommended amounts of sugar/sweeteners (32 g in Germany, 30 g in India, & 60 g in Uruguay)

^**d**^These diets also include recommended amounts of discretionary calories (308 in the Netherlands, 270 cal in US, and 290 in US Vegetarian)

^**e**^Pulses only

^**f**^Eggs, fish and seafood, meat

^**g**^Includes potatoes

^**h**^Includes roots and tubers

^**i**^Includes legumes

^**j**^ Fruit and vegetables form one group in Uruguay, so the 455 g is the recommendation for the combined group

Table 2 displays our approach for calculating the greenhouse gas emissions associated with the recommendation for one food group, fruits, in one country, the US. Overall, the GHGE from this recommendation of 2 c/d is 0.16 kg CO_2_-eq/d. As can be seen from the table, this is a weighted average of GHGE for different fruits, with the weights determined by apparent consumption data from FAO’s food balance sheets.

**Table 2:** Calculation of greenhouse gas emissions in the US daily recommendation for fruit^**a**^

FAO Food BalanceSheet Commodity	Total of FruitRecommended(cups/d)^**b**^	Apparentconsumptionproportion^**c**^	Conversion factor(cups to kg)^**d**^	GHGE impact factor(kg CO_2_-eq/kg)^**e**^	GHGE(kg CO_2_-eq)
Apples and products		0.178	0.110	0.228	0.009
Bananas		0.113	0.150	0.374	0.013
Citrus, other		0.002	0.185	0.438	0.000
Dates		0.001	0.075	2.024	0.000
Grapefruits and products		0.017	0.210	1.210	0.008
Grapes and products		0.085	0.120	0.478	0.010
Lemons, limes, & products		0.091	0.210	0.517	0.020
Oranges, tangerines, mandarins, & products		0.231	0.185	0.347	0.030
Pineapples and products		0.061	0.165	0.914	0.018
Fruits, other		0.222	0.196	0.652	0.057
TOTAL	2	1.000			0.165

^**a**^This is an average of the GHGE in various fruit commodities, weighted by the apparent US consumption proportion of each commodity, which was calculated from FAO food balance sheet data. For each row, columns 3–5 are multiplied by each other and by the total recommendation for fruit (2 cups/d) to arrive at the contribution of that commodity to the total GHGE. All rows in the final column are summed to arrive at the overall total of GHGE for the fruit recommendation

^**b**^From US Dietary Guidelines for Americans for a 2000 kcal diet

^**c**^Authors’ calculations from FAO food balance sheet data [46]

^**d**^From various sources [45, 48–50]

^**e**^From dataFIELD [45]

The daily GHGE attributed to the recommended diet of each country, broken down by food group, is displayed in Table 3. For the protein food recommendations, this ranges from 0.03 kg CO_2_-eq/d in India to 1.84 kg CO_2_-eq/d in the United States. For dairy, this ranges from 0.17 kg CO_2_-eq/d in Oman to 1.10 kg CO_2_-eq/d in the United States. The total GHGE that could be attributed to eating the country’s recommended diet ranges from 0.86 kg CO_2_-eq/d in India to 3.83 kg CO_2_-eq/d in the United States. The US vegetarian guideline is the third lowest in terms of GHGE (1.80 kg CO_2_-eq/d), though much higher than India’s, due largely to the differences in dairy recommendations.

**Table 3:** Greenhouse gas emissions (kg of CO2-eq) of daily food group recommendations for a 2000-kcal diet pattern by country^**a**^

	Protein Foods	Dairy	Grains	Fruit	Vegetables	Oils/fats	Total
Germany	0.98	0.81	0.05	0.12	0.20	0.09	2.25^**b**^
India	0.03	0.41	0.11	0.05	0.17	0.08	0.86^**b**^
Oman	1.24	0.17	0.20	0.39	0.27	0.26	2.53
The Netherlands	1.12	0.89	0.08	0.08	0.12	0.15	2.86^**c**^
Thailand	0.63	0.33	0.31	0.47	0.09	N/A	1.83
United States	1.84	1.10	0.02	0.16	0.16	0.03	3.83^**c**^
US Vegetarian	0.06	1.10	0.02	0.16	0.16	0.03	1.80^**c**^
Uruguay	1.50	0.65	0.03	0.18^**d**^		0.04	2.42^**b**^
EAT-Lancet	0.79	0.30	0.03	0.08	0.12	0.05	1.36

^**a**^Calculation of greenhouse gas emissions for each food group requires more specificity than is provided in any country’s recommendations. This specificity comes from using FAO food balance sheet data [46], which provides the proportion of specific foods consumed within a food group and is based on each country’s food availability data

^**b**^Total includes GHGE of daily recommended amounts of sugar and sweeteners

^**c**^Total includes GHGE of daily recommended discretionary calories

^**d**^Fruit and vegetables form one group in Uruguay, so 0.18 refers to the emissions for the combined group

The GHGE results described above are based on using each country’s FBDG as well as its consumption patterns, calculated from FAO food balance sheet data. To examine the sole impact of the recommendations, the GHGE attributable to the FBDG were recalculated assuming the specific foods for each country’s guidelines all used the same apparent consumption pattern, namely that of the US. As seen in Table 4, after controlling for the details of the consumption pattern, the US dietary recommendations, at 3.83 kg CO_2_-eq/d, still have the highest carbon footprint of any of the seven countries. The footprint of the US FBDG is about 1.2 times that of the Netherlands’, about 1.5 times that of Germany’s, and about 5.2 times that of India’s.

**Table 4:** Greenhouse gas emissions (kg of CO_2_-eq) of daily food group recommendations for a 2000-kcal diet pattern by country, controlling for apparent consumption with US food balance sheet data^**a**^

	Protein Foods	Dairy	Grains	Fruit	Vegetables	Oils/fats	Total	US Ratio^**c**^
Germany	1.37	0.81	0.05	0.12	0.18	0.05	2.61^**b**^	1.47
India	0.02	0.41	0.05	0.05	0.18	0.03	0.74^**b**^	5.19
Oman	1.14	0.18	0.44	0.29	0.18	0.05	2.29	1.68
The Netherlands	1.51	0.90	0.08	0.09	0.11	0.04	3.22^**d**^	1.19
Thailand	1.61	0.37	0.09	0.47	0.09	N/A	2.63	1.46
United States	1.84	1.10	0.02	0.16	0.16	0.03	3.83^**d**^	1.00
US Vegetarian	0.06	1.10	0.02	0.16	0.16	0.03	1.80^**d**^	2.13
Uruguay	1.24	0.70	0.03	0.19^**e**^		0.02	2.22^**b**^	1.73
EAT-Lancet	0.79	0.30	0.03	0.08	0.12	0.05	1.36	2.82

^**a**^FAO food balance sheet data [46] for the US is used to provide the proportion of specific foods consumed within each food group for all countries listed. Thus, any differences between countries are due solely to the recommendations themselves

^**b**^Total includes GHGE of daily recommended amounts of sugars or sweeteners

^**c**^US ratio is the ratio of the total GHGE for the US recommended 2000-kcal diet divided by the total GHGE for each country’s recommended diet

^**d**^Total includes GHGE of daily recommended discretionary calories

^**e**^Fruit and vegetables form one group in Uruguay, so 0.19 refers to the emissions for the combined group

## Discussion

This study found both similarities and differences in the quantitative recommendations of the FBDG of our study countries. The principal similarity is the presence of six common food groups – protein foods, dairy, grains, fruits, vegetables, and oils/fats – although Thailand’s FBDG does not include an oils/fats groups and Uruguay’s FBDG combines fruits and vegetables into one group. The principal difference among the FBDG is the wide range of daily recommended amounts for each group. Recommendations for protein foods in the US are more than twice those for India, while dairy recommendations in the US are six times those in Oman and three times those in Thailand. There are also differences in which specific foods are included in this group, with the United States and Thailand recommending a full spectrum of plant and animal protein foods, whereas India recommends just plant proteins and the US vegetarian FBDG recommends plant foods, as well as dairy and eggs. These results are consistent with previous work that demonstrated a number of differences among the countries’ FBDG [16].

There are also sizable differences in the carbon footprints of recommended diets. Our results ranged from a low for India at 0.86 kg CO_2_-eq/d to a high for the United States of 3.83 kg CO_2_-eq/d. The carbon footprint of the basic US guideline is over twice that of the US vegetarian guideline. These results are broadly consistent with other literature on this topic. For example, Blackstone and colleagues found that the US FBDG was 24.8 kg C0_2_-eq/wk., or 3.54 kg CO_2_-eq/d, which is about 8% lower than our estimate [51]. This could easily be attributable to the differences in emission factors or consumption factors used in the two studies. More importantly, both studies found that the main US guideline was about twice that of the vegetarian in terms of GHGE. Our results on the carbon footprint of Netherlands FBDG is substantially lower than a previous calculation, 2.86 vs. 4.0 kg CO_2_-eq/2000 kcal [27]. This is likely due to differences in boundary conditions, as we focused on food production, and the Dutch study used a total life cycle (cradle to plate) approach.

There are three main factors that result in differences in the GHGE of the recommended diets we studied. First, the daily recommended amounts of each food group influence the GHGE of a country’s recommended diet. This is particularly true for the protein and dairy food groups as animal foods have larger carbon footprints than plant-based foods, so larger protein foods and dairy recommendations, to the extent that they are met by animal foods, particularly from ruminants, contribute to larger GHGE attributable to the total diet [45]. The US daily recommendation for dairy is three times that of Thailand’s, as is the corresponding GHGE from that group.

Second, the inclusion of specific recommendations in these groups also greatly affects the GHGE attributable to each recommended diet. For example, protein food recommendations in Germany and Uruguay only include animal proteins, India’s only includes plant proteins, and others include a mixture of animal and plant proteins. The EAT-Lancet guideline amount for the protein food group is greater than that of the US FBDG, but over half of this is allocated to plant proteins, resulting in an overall GHGE for the protein foods recommendation that is about half of the US recommendation. The US Vegetarian FBDG is almost identical in recommendations to the main US guidelines, except for the protein group – which recommends legumes, soy, nuts, and seeds, as well as eggs – resulting in an overall carbon footprint that is less than half. In the US FBDG, fortified soymilk is listed in the dairy products group, but it is not well publicized, relegated to a footnote at the end of the recommendations table [40]. Suggesting a specific recommendation for plant-based alternatives to dairy, as was done in the vegan adaptation of the 2010 DGA [52], could lower the GHGE from the dairy group substantially. For example, previously we found that the GHGE from the production of cow milk (1.32 kg CO_2_-eq/kg) was over five times that of soy milk (0.26 kg CO_2_-eq/kg) [45].

Third, the apparent consumption pattern in each country, which provides specificity to our calculation of the GHGE attributable to each food group recommendation, also influences our results on a country’s FBDG carbon footprint. Foods with the greatest global warming impact factors, such as those from ruminant animals (e.g. beef), have the potential to drive up the GHGE that could be attributed to a recommended diet if they are consumed in large proportions relative to the other foods included in the same food group. For example, apparent consumption of beef, mutton, and lamb in Uruguay accounts for 31% of protein foods, whereas in Germany it is only 16%. Thus, our calculated GHGE attributed to Uruguay’s protein food recommendation is 53% higher than that of Germany, despite the fact that both countries’ quantity recommendations for protein foods are about the same.

This is a necessary limitation to determining the carbon footprint of a country’s FBDG, since most recommendations, other than, for example, the EAT-Lancet guidelines, do not provide specific quantities of foods (e.g. beef) within a group (e.g. protein foods). Moreover, some FBDG assume “discretionary calories” will be consumed as part of their recommendations, and do not specify the foods that makeup these calories. Our approach – the use of FAO food balance sheet data to create apparent consumption patterns in each country – provides a realistic representation of national food consumption patterns within each food group, using a consistent data source across all countries, and has been used by others [29, 30]. We also used this approach to assign the GHGE for discretionary calories, assuming the foods that make up these additional calories would be similar to the rest of the recommended diet. Though this might under or overestimate the footprint of recommended diets, the ambiguity of the FBDG themselves requires this uncertainty.

Still, this challenges our ability to compare the FBDG as a policy tool across countries as it relates to environmental sustainability, since our Table 3 results are determined by both the FBDG *and* the country’s existing consumption pattern. We addressed this by controlling for countries’ dietary patterns, using the same pattern of apparent consumption, that of the US, for all countries studied here. This allowed us to better estimate the carbon footprints of different guidelines for purposes of comparing these guidelines between countries.

Another limitation of this study is that it only considers a single environmental impact of diets, GHGE, and uses one set of impact factors for all countries studied. There are other environmental impacts of food, such as land and water use, that should be considered when evaluating the overall impact of a diet, since they could yield different results on the comparisons of environmental impacts of the FBDG studied here. Unfortunately, because of the limitations of current data availability, it is not possible to use specific country-level impact factors for the countries studied here, not even for GHGE.

A third potential limitation is that this study is restricted to the daily quantitative recommendations of seven countries’ FBDG, which, of course, limits its generalizability. Our goal, however, has not been to present generalizable results about all countries’ dietary guidance policies. Rather, we have sought to illustrate a range of such dietary guidance policy options and the potential implications of those options for greenhouse gas emissions, as well as address the anomaly found in previous research that dietary shifts toward a country’s FBDG reduces GHGE except in the US. The seven countries that were included in this study were purposively selected to accomplish these goals.

## Conclusions

We found the carbon footprint of the US recommendation is higher than that of all other countries studied here, even after controlling for country-level consumption patterns. This includes other high-income countries in Europe, like the Netherlands and Germany, where the US recommendation is 19% and 47% greater, respectively. The footprint of the US recommendation is over twice that of plant-forward recommendations, like that of EAT-Lancet, the US Vegetarian, or India’s guidelines. These differences are due to the higher quantities of protein and dairy food recommendations in the US FBDG, as well as the types of protein foods recommended.

These findings hold insights for future development of FBDG. To lower the carbon footprint of their recommended diets, countries with large impacts attributable to the protein foods or dairy groups could either reduce the recommended daily amount of these groups or create recommendations for sub-groups of plant proteins within the larger protein foods group, as Oman does with its recommendation for legumes, or the EAT-Lancet guideline does with its specific limits on ruminant consumption and recommendations for various plant protein foods. Similar suggestions, such as reducing the recommended daily amount or encouraging the consumption of plant-based substitutes, could be implemented to lower the carbon footprint of recommended diets for countries with large impacts coming from the dairy group.

A number of countries have discussed sustainability in their dietary guidance materials [36], and two of the FBDG we studied here – the Netherlands and the EAT-Lancet – include quantitative recommendations based on both nutrition and sustainability considerations [38, 44]. FBDG are important policy tools that influence other national food and nutrition policies, such as school and other institutional-based feeding recommendations. Finding and incorporating a reasonable balance between nutrition and sustainability into national FBDG has the potential to not only influence individuals’ diets and carbon footprints [53], but to also impact national food policies that shape many elements of national and global food systems. Thus, the inclusion of sustainability considerations and recommendations in dietary guidelines will be an important part of future policy activities to reduce the environmental impact of the food and agriculture sector. This paper provides a starting point for considering a range of potential impacts on global warming from existing dietary recommendations.

## Supplementary Information

**Additional file 1: Table S1.** Daily recommended amounts of food groups by country as presented in each country’s food-based dietary guideline.

**Additional file 2. **GHGE calculations of food based dietary guidelines in the 7-country study.

## Data Availability

The datasets analysed in the current study are available as follows: FAO Food Balance Sheet Data: http://www.fao.org/faostat/en/#data/FBS dataFIELD: http://css.umich.edu/page/datafield

## Abbreviations

CO_2_-eq: Carbon-dioxide equivalents
DGA: Dietary Guidelines for Americans
FAO: United Nations Food and Agriculture Organization
FBDG: Food-based dietary guidelines
GHGE: Greenhouse gas emissions
OECD: Organization for Economic Co-operation and Development
