# Supplementary material for: The carbon footprint of dietary guidelines around the world: a seven country modeling study
Source: Nutr J. 2021 Mar 1;20:15. doi: 10.1186/s12937-021-00669-6 (PMC7923667; doi:10.1186/s12937-021-00669-6)
Supplement: Supplementary file 1 — Additional file 1: Table S1. Daily recommended amounts of food groups by country as presented in each country’s food-based dietary guideline. [file 12937_2021_669_MOESM1_ESM.docx]

**Table S1.** Daily recommended amounts of food groups by country as presented in each country’s food-based dietary guideline

|  | Protein foods | Dairy | Grains | Fruit | Vegetables | Oils/fats |
| --- | --- | --- | --- | --- | --- | --- |
| Germany**^a^** | - 64 g meat - 26 g fish & seafood - 9 g eggs | - 55 g high fat (e.g. cheese) - 225 g low fat (e.g. milk, yogurt) | - 362 g | - 250 g | - 400 g includes roots and tubers - 112 g potato products | - 24 g vegetal oils & margarine - 11 g butter |
| India**^a^** | - 75 g pulses | - 300 ml | - 330 g | - 100 g | - 200 g roots & tubers - 100 g green leafy - 200 g other | - 25 g |
| Oman | - 80 g nuts, eggs, fish, meat - 0.5 c legumes | - 0.5 c | - 3.5 c includes potatoes | - 3.5 c | - 3 c | - 55.5 g |
| The Netherlands**^b^** | - 25 g nuts and seeds - 100 g/wk fish and shellfish - 150 g/wk legumes - 300 g/wk red meat - 200 g/wk white meat - 125 g/wk eggs | - 375 g milk and dairy products - 40 g cheese | - 270 g cereal products and potatoes - 157.5 g bread | - 200 g | - 250 g | - 40 g spreadable and cooking fats |
| Thailand | - 135 g legumes, nuts/seeds, eggs, fish & seafood, meat | - 1 c | - 600 g includes roots, tubers | - 4 pieces | - 200 g | N/A |
| United States**^b^** | - 8 oz-eq/wk seafood - 26 oz-eq/wk meats, poultry, eggs - 5 oz-eq/wk nuts, seeds, soy products | - 3 c | - 6 ounce-equivalents | - 2 c | - 1.5 c/wk dark green - 5.5 c/wk red & orange - 1.5 c/wk beans & peas - 5 c/wk starchy - 4 c/wk other | - 27 g |
| United States Vegetarian**^b^** | - 3 oz-eq/wk egg - 6 oz-eq/wk legumes - 8 oz-eq/wk soy - 7 oz-eq/wk nuts & seeds | - 3 c | - 6.5 ounce-equivalents | - 2 c | - 1.5 c/wk dark green - 5.5 c/wk red & orange - 1.5 c/wk beans & peas - 5 c/wk starchy - 4 c/wk other | - 27 g |
| Uruguay**^a^** | - 100 g eggs, fish & seafood, meat | - 500 ml | - 250 g includes legumes | 500 g | | - 30 ml |
| EAT-Lancet | - 7 g beef/lamb - 7 g pork - 29 g poultry - 13 g eggs - 28 g fish - 50 g dry beans - 25 g soy foods - 25 g peanuts - 25 g tree nuts | - 250 g whole milk or derivative equivalents | - 232 g | - 200 g | - 100 g dark green - 100 g red and orange - 100 g other - 50 g tubers or starch | - 6.8 g palm oil - 40 g unsaturated oils - 5 g lard or tallow |

**^a^** Daily recommendations also include recommended amounts of sugar/sweeteners (32g in Germany, 30g in India, & 60g in Uruguay).

**^b^** Daily recommendations also include recommended amounts of discretionary calories (308 in the Netherlands, 270 calories in US, & 290 in US Vegetarian). The amount for the Netherlands is calculated by taking the calorie content of the recommended diet for adult women, which is 1745, and dividing by .85, since the recommended foods meet 85% of energy needs (Brink et al, 2018 – reference #38). This totals 2053, so discretionary calories are the remainder of 308. The amounts for the US are listed in the FBDG column for 2000 kcal.
